# Looking at Sex Differences in Gambling Disorder: The Predictive Role of the Early Abandonment Schema, Gambling Motives and Alexithymia in Depression

**DOI:** 10.1007/s10899-023-10251-w

**Published:** 2023-09-21

**Authors:** Ana Estévez, Laura Macía, Patricia Macía

**Affiliations:** 1 Psychology Department, Faculty of Health Sciences, University of Deusto, Apartado 1, 48080 Bilbao, Spain; 2 Department of Basic Psychological Processes and Their Development, University of the Basque Country/Euskal Herriko Unibertsitatea (UPV/EHU), 20018 Donostia-San Sebastián, Spain

**Keywords:** Gambling, Women, Abandonment, Gambling motives, Alexithymia, Depression

## Abstract

A growing body of research indicates differences between men and women with gambling disorder. However, although alexithymia, gambling motives or abandonment have been associated with GD, women’s clinical profile remains unexplored. This study aims, firstly, to explore mean differences in gambling motives (i.e. enhancement, coping and social [SOC]), the early maladaptive abandonment schema, alexithymia and depression in women and men with GD. Secondly, to analyse the correlation between the aforementioned variables as a function of sex. Thirdly, to examine the predictive role of gambling motives, early abandonment schema and alexithymia in depression as a function of sex. Lastly, to analyse the mediating role of COP between the early maladaptive abandonment schema and depression in women with GD, and the mediating role of ENH and the early maladaptive abandonment schema between alexithymia and depression in men with GD. The sample comprised 108 adults with GD diagnosis, of whom 60 were women and 48 were men. Regression and mediation analyses were carried out to explore possible sex differences in GD, through the SPSS programme. Results showed that depressive symptoms are predicted by alexithymia in men with GD and by the early maladaptive abandonment schema in women with GD. Regarding mediation analyses, the results suggest that COP mediated the relationship between early abandonment schema and depressive symptoms in females; and ENH and abandonment schema mediated the relationship between alexithymia and depressive symptoms in males. These results provide evidence of the relevance of considering sex differences when establishing therapeutic strategies in GD rehabilitation.

## Introduction

Gambling disorder (GD) is a behavioural addiction characterised by persistent, uncontrollable and maladaptive patterns of gambling activity, despite the clinically significant consequences it generates (loss of work, deterioration of important personal relationships, indebtedness, emotional disorders, etc.) (American Psychiatric Association [APA], 2013). According to a current meta-analysis, the prevalence of gambling disorder is estimated to be 1.29% in adults (Gabellini et al., 2023).Concerning GD prevalence as a function of sex, a ratio of 2:8 of women and men has been found (Merkouris et al., 2016). Although there is still limited gender-specific literature on GD, studies have reported sex-related differences in terms of psychopathological profile, gambling behaviour patterns, associated consequences or pathogenesis of GD (Gavriel-Fried et al., 2019; Jiménez-Murcia et al., 2020; Macía et al., 2023). In this regard, for example, it has been observed that different emotions are involved in women and men with gambling problems. While men tend to feel a mixture of anger and excitement, women often suffer from shame and guilt (McCormack et al., 2014). In this sense, guilt and shame in women with GD have been explained by the fact that it is more stigmatising for women than for men to suffer from this disorder (Del Rosal et al., 2020; Macía & Estévez, 2023; Soares & Carvalho, 2009). This, in turn, may also help to justify the under-diagnosis of female gamblers and the fact that they seek less therapeutic help (Baño et al., 2021; Belloch et al., 2020; Lamas et al., 2018).

Gambling motives are one of the main aetiological factors of GD, explaining an individual's vulnerability to developing an addictive behaviour in both young and adult populations (Jauregui et al., 2020). The literature suggests that gambling motives predict gambling behaviour, as well as its frequency, variety, severity or associated gambling problems (Barrada et al., 2019; Grande-Gosende et al., 2019; Hagfors et al., 2023; Macía et al., 2022; Stewart & Zack, 2008). According to the model proposed by Stewart and Zack (2008), there are three main motives for gambling: (a) enhancement motives (ENH; “*Because it’s exciting*”); (b) coping motives (COP; “*Because it helps when you are feeling nervous or depressed*”); and (c) social motives (SOC; “*Because it makes a social gathering more enjoyable*”). ENH and COP aim to regulate emotional states and have been associated with GD and its severity to a greater extent than SOC (Dechant & Ellery, 2011; Grande-Gosende et al., 2019). Men are more likely to gamble to demonstrate skills, compete or for financial motives. Even being part of a betting community can have social benefits for men (Lamont & Hing, 2018). Likewise, ENH has been associated with sensation-seeking and GD severity in men (Jauregui et al., 2018; Stewart & Zack, 2008). By contrast, women with GD are more likely to use gambling as a coping mechanism to deal with stressful life events and negative emotions (Lelonek-Kuleta, 2021). Previous literature has identified COP as the strongest motive to predict GD severity and associated damage (Schellenberg et al., 2016). This is particularly noteworthy in view of the fact that women are more likely to report COP than men (Macía & Estévez, 2021; Macía et al., 2022).

Early Maladaptive Schemas (EMS) are defined as internalised and stable patterns acquired through early affective experiences or negative interactions with representative figures in childhood, influencing how we feel, think, behave and relate with others throughout life, including adulthood (Estévez, 2013; Young et al., 2013). Relationships between EMS and mental health disorders have widely been studied, including substance and behavioural addictions, such as GD, interpersonal problems or depression, among others (Aloi et al., 2020; Bishop et al., 2022; Shorey et al., 2012). Of note is that women tend to experience higher levels of EMS than men (Bilge & Balaban, 2021; Janson et al., 2019). One of the dimensions of EMS related to GD is “Disconnection/Rejection”, which includes the schema of abandonment (Efrati et al., 2022). The abandonment schema refers to the expectation or belief that close people will inevitably leave or not be available to provide emotional support (Rafaeli et al., 2010; Young et al., 2013). Furthermore, the Disconnection/Rejection dimension of EMS is closely related not only to feelings of abandonment but also to emotional deprivation and feelings of guilt and shame, which, as mentioned, are more frequent in female gamblers (van Wijk-Herbrink et al., 2018). In turn, previous evidence suggests that disconnection/rejection is related to both internalising (i.e., depression) and externalising behavioural problems (i.e., GD), and that coping responses mediate these effects (D'Rozario & Pilkington, 2022; van Wijk-Herbrink et al., 2018). However, the literature exploring GD and EMS is still very scarce, and little is known about possible sex differences.

On the other hand, Rogier and Velotti (2018) point out that some people with GD tend to fail to identify their emotions due to deficits in emotional awareness, difficulties in accepting affective states and alexithymic personality traits. Alexithymia is characterised by a low capacity to identify, analyse, describe and differentiate one’s own and other people's emotional states (Brewer et al., 2016). Previous evidence shows that alexithymic traits are predictive of GD, and that alexithymia is also associated with problem gambling behaviours and increased severity of gambling symptoms (Bonnaire et al., 2017; Elmas et al., 2017; Estévez et al., 2020; Marchetti et al., 2019). Alexithymia has also been related to depression (Hemming et al., 2019; Macía et al., 2023), hopelessness (Serafini et al., 2020), gambling motives (Marchica et al., 2020) and EMS (Aust et al., 2013). In fact, prior research has noted that people with alexithymia scored higher on EMS and depression than non-alexithymic people, and that the co-occurrence of the aforementioned three variables (alexithymia, EMS and depression) appears to worsen the psychopathological condition (Saariaho et al., 2015).

For its part, depression has been associated with early cognitive schemas (Nicol et al., 2020), abandonment (Ahmadpanah et al., 2017), and GD (Richard et al., 2020). In this line, several empirical studies have supported differences in depressive symptomatology between men and women with GD (Macía et al., 2023; Salk et al., 2017; Sundqvist & Rosendahl, 2019). Female gamblers appear to be more likely to report depressive symptoms, as well as a previous experience of an affective disorder (Bonnaire et al., 2017; Grant et al., 2012). In this vein, numerous studies have identified insecure attachment as a core factor in substance and non-substance addictions, emotional disorders, alexithymia, difficulties in emotional regulation, EMS, or greater impact of traumatic experiences such as abandonment (Estévez et al., 2017, 2020; Keough et al., 2018; Oshri et al., 2015; Zdankiewicz-Ścigała & Ścigała, 2018). Furthermore, insecure attachment experiences have been observed to predict elevated depression, increased gambling motives and increased severity of GD (Estévez et al., 2020; Macía et al., 2022). The nature of the association between GD, depression, EMS, alexithymia and gambling motives, however, remains unclear. Although it is known that there might be a relationship between these variables, they have hardly been studied interrelatedly. To our knowledge, there are no studies exploring alexithymia, EMS and gambling motives as predictors of depression in people with GD. Moreover, a growing body of research indicates differences between men and women with GD. Further research is needed to deepen knowledge about sex differences, especially concerning women with GD.

Therefore, the objectives of this study are the following: (1) to compare mean differences for all study variables (gambling motives, maladaptive abandonment schema, alexithymia and depression) in a sample of people with GD as a function of sex; (2) to analyse the correlation of the aforementioned variables as a function of sex; (3) to examine the predictive role of gambling motives (ENH, COP and SOC), the early maladaptive abandonment schema and alexithymia in depression as a function of sex, (4) to analyse the mediating role of COP between the early maladaptive abandonment schema and depression in women with GD; and (5) to analyse the mediating role of ENH and the early maladaptive abandonment schema between alexithymia and depression in men with GD.

## Method

### Participants

The sample comprised 108 participants with GD diagnosis, of whom 60 (55.6%) were women and 48 (44.4%) were men. The sample was collected by convenience sampling method, since it is a sample that is difficult to locate (men and women with GD diagnosis). All participants were over 18 years. Inclusion criteria for participating in the study were: (a) having attended or to be currently undergoing treatment in an assistance centre specialised in GD; and (b) scoring as a gambler in the SOGS questionnaire (Lesieur & Blume, 1987). Regarding the first criteria, participants were out-patients recruited through GD treatment associations belonging to the FEJAR. FEJAR is a Spanish organisation aimed at rehabilitating GD, which comprises multiple associations throughout the country with the same purpose. Regarding the second criteria, according to the SOGS tool (Spanish validation), scores of 4 or more indicate “potential gambling risk”, scores between 2 and 3 indicate “gambling risk”, while scores between 0 and 1 indicate “no gambling risk” (Echeburúa et al., 1994). Therefore, in order to be part of the sample, it was necessary that they also scored 4 or more on the SOGS. Otherwise, they were excluded from the sample of this study (see more information in the procedure section).

In Table 1, sociodemographic characteristics are described. Participants’ mean age ranged from 21 to 79 years (*M* = 45.63, *SD* = 13.06). Regarding educational level, most of them had vocational training (38%) or primary school (24%), respectively; and were employed (55.6%). Regarding civil status, almost 39% of the participants were single, whereas 38% were married or lived with a partner; another 15.7% were separated or divorced, and 4.6% were widowed.

**Table 1:** Sociodemographic analysis in women and men with gambling disorder (n = 108)

Sociodemographic variables		n	%
Sex	Women	60	55.6
	Men	48	44.4
Ed. Level	Primary school	24	22.2
	Secondary school	8	7.4
	Bachelor	9	8.3
	Professional training	41	38.0
	University	19	17.6
	Others	7	14.6
Employment	Paid work	60	55.6
	Unemployed	16	14.8
	Retired	15	13.9
	Disabled	4	3.7
	Others	13	12
Civil status	Single	42	38.9
	Married, in couple	41	38.0
	Separated, divorced	17	15.7
	Widower	5	4.6
	Others	3	2.8

M = mean, SD = standard deviation

### Instruments

#### Early Maladaptive Abandonment Schema

*Young Schema Questionnaire—Short Form* (YSQ-SF; Young & Brown, 1994) in its Spanish version was used to measure the early maladaptive *abandonment* schema (Calvete et al., 2005). The YSQ-SF evaluates early dysfunctional schemas proposed by Young, including 60 items that assess 18 dysfunctional schemas divided into five domains: Disconnection and rejection (emotional deprivation, abandonment, abuse/distrust, social isolation, imperfection), Impaired autonomy (failure, dependence/incompetence, vulnerability to danger, attachment/ entanglement), Orientation to others (subjugation, self-sacrifice, recognition-seeking), Impaired limits (grandiosity, insufficient self-control) and Excessive vigilance and inhibition (emotional inhibition, unattainable goals, negativity and self-punitiveness). In the present study, we used the abandonment schema, referring to the tendency to believe that significant others will not provide needed emotional support or protection because they will abandon one for someone better. The SQ-SF has a Likert-format scale with six response options ranging from 1 (*Completely false*) to 6 (*It describes me perfectly*). Although the YSQ-SF has not been validated in samples with GD, previous literature points to positive and significant correlations between early abandonment schema and GD (Helpful, 2017). Regarding internal consistency, the instrument showed a good index for the overall scale (over 0.74 for all subscales), and in the present study, Cronbach’s alpha was 0.94 for all subscales and 0.84 for the Abandonment schema scale.

#### Alexithymia

The *Toronto Alexithymia Scale-20* (TAS-20; Bagby et al., 1994a, b) in its Spanish version (Martínez-Sánchez, 1996) was used to assess participants’ alexithymia. The scale contains 20 items divided into three main dimensions: (1) Difficulty Identifying Feelings, referring to trouble in identifying emotions, often confusing them with physical symptoms; (2) Difficulty Describing Feelings, which refers to the inability to communicate feelings and use emotional vocabulary; and (3) Externally-Oriented Thinking, in which individuals tend to neglect their inner emotional states, and are oriented toward concrete and external details. The scale is rated on a 6-point Likert scale that ranges from 0 (*Strongly disagree*) to 5 (*Strongly agree*). The TAS-20 has not been validated in people with GD, however, it has been widely used, with good psychometric indicators, to assess the influence of alexithymia on disorders such as gambling, substance abuse, depression or psychosomatic disorders. Internal consistency was very good for the overall scale (α = 0.83 for the original scale and 0.81 for the Spanish adaptation), and Cronbach’s alpha in the current study was 0.84.

#### Gambling Motives

*Gambling Motives Questionnaire* (GMQ; Stewart & Zack, 2008) was used in its Spanish version (Jáuregui et al., 2018) to assess 15 reasons why people gamble. The GMQ questionnaire is divided into three main factors comprising five items each: (1) Enhancement Motives (ENH): referring to positive internal reinforcement to increase positive emotions (e.g., *To get "intense" feeling*); (2) Coping Motives (COP): alluding to negative internal reinforcement, to avoid or lessen negative emotions (e.g., "*Because it helps you when you feel nervous or depressed*"); (3) Social Motives (SOC): referring to positive external reinforcement, mainly social affiliation (e.g., "*Because it's what most of your friends do when they get together*"). Each item is an adaptation of the Drinking Motive Questionnaire (Cooper et al., 1992). The scale is rated on a four-point Likert scale with response options ranging from 1 (*never/almost never*) to 4 (*almost always*). Regarding psychometric properties, all subscales presented good internal consistency (α > 0.80) in the original study and the Spanish version (between 0.71 and 0.85). In the current study, Cronbach's alpha ranged from 0.84 to 0.87.

#### Depressive Symptomatology

*Symptom Checklist-90-Revised* (SCL-90-R; Derogatis, 1977, adapted to Spanish by De las Cuevas et al., 1991). The SCL-90-R is a self-administered questionnaire that evaluates 90 symptoms classified into nine psychopathology symptoms and three global distress indexes. In this specific study, we used the psychopathological symptom of Depression, composed of 13 items that include a great variety of signs and symptoms characteristic of depressive disorder (e.g., despondency, anhedonia, self-destructive thoughts, etc.). Severity of each symptom is rated on a 5-point Likert scale ranging from 0 (*no symptom-related distress*) to 4 (*maximum distress*). Regarding the psychometric properties of the scale, the internal consistency was very good for the overall scale (higher than 0.70 for all subscales). In the present study, Cronbach’s alpha was 0.94 for the Depression subscale.

### Procedure

This study used a cross-sectional research design. A total of 108 participants with GD were recruited through GD treatment associations belonging to FEJAR (Spanish Federation of Rehabilitated Gamblers). Participants completed the questionnaires both online and offline (pencil and paper). According to Herrero-Fernández (2015), the application method of questionnaires (pencil and paper vs. online) does not affect the results obtained. The online questionnaire could be accessed via the link to the questionnaire or a QR code. On the other hand, participants who answered the questionnaire in offline format, completed the questionnaire in treatment centres for the rehabilitation of GD.

Participants had to read the study information and provide informed consent to access the questionnaire. They also had to be over 18 years old. Completing the questionnaire required around 40 min. Before completing the questionnaire, participants received general information about the main objectives of the research study. It was made clear that there were no right or wrong answers, and they could contact the main researcher by mail if they needed further information about the study. Confidentiality, anonymity, and voluntary participation were ensured for all participants, who did not receive any compensation for participating.

The research obtained the ethics committee’s approval from the Institutional Review Board of the first author’s university. This study was performed in line with the principles of the Declaration of Helsinki.

### Statistical Analysis

Firstly, descriptive analysis and mean differences by sex through Student´s *t* were calculated for variables of gambling motives, abandonment early maladaptive schema, alexithymia and depression. Secondly, bivariate correlations were conducted to explore the relationship between gambling motives, the early maladaptive abandonment schema, alexithymia and depression in women and men. Thirdly, multiple regression analysis was carried out to analyse the predictive effect of gambling motives, abandonment schema and alexithymia on depression (as an outcome variable) in men and women separately. These analyses were conducted through the “Intro”/”Enter” method (that is, inserting all the predictor variables at the same time).

Fourthly, in light of the differential results obtained for both sexes in the regression analyses, a mediation analysis was conducted to explore whether, on the one hand, in the case of women, COP (as a mediator—M) mediated the relationship between abandonment schema (as an independent variable—IV) and depression (as a dependent variable—DV). On the other hand, in the case of men, whether abandonment schema and ENH (as mediator variables—M) mediated the relationship between alexithymia (as an independent variable—IV) and depression (as a dependent variable—DV).

All these statistical analyses were performed through the SPSS-22 programme (Statistical Package for the Social Sciences, 2013). Specifically, the PROCESS programme of SPSS by Hayes and Preacher (2013) was used for the mediation analysis.

## Results

### Descriptive Analysis and Mean Differences

Firstly, descriptive analysis and mean differences were calculated by sex differences for gambling motives, the early maladaptive abandonment schema, alexithymia and depression in women and men with GD (Table 2). Results showed that men and women differed in the level of alexithymia (*t*_(46,45)_ = − 1.887,  *p* = 0.033), being higher in women (*M* = 70.67, *SD* = 11.69) than in men (*M* = 63.87, *SD* = 13.42).

**Table 2:** Mean differences by sex in gambling motives, the early abandonment schema, alexithymia and depression

	Men with GD (n = 48)	Women with GD (n = 60)	
	M (DT)	M (DT)	t _(*df*)_
GMQ_social	6.68(1.84)	6.75(2.33)	− 0.164(101,100)
GMQ_coping	11.41(3.61)	12.70(4.49)	− 1.592(98,97)
GMQ_enhance	11.76(4.04)	10.98(4.53)	0.991(99,98)
Abandonment schema	13.84(6.68)	13.83(7.27)	0.007(92,85)
TAS-20	63.81(13.42)	70.67(11.69)	− 1.887(46,45)*
Depression	31.88(13.16)	32.55(14.19)	− 0.236(96,93)

M = mean, SD = standard deviation; t = Student´s; df = freedom degrees; GMQ = Gambling Motives Questionnaire; TAS-20 = Toronto Alexithymia Scale-20. * = *p* < 0.05

### Bivariate Correlation Analysis

Bivariate correlations were calculated between variables of gambling motives, the early maladaptive abandonment schema, alexithymia and depression in men with GD (Table 3). ENH (*r* = − 0.35, *p* < 0.001), early maladaptive abandonment schema (*r* = 0.72, *p* < 0.001) and alexithymia (*r* = 0.69, *p* < 0.001) were significantly positively correlated with depression. In addition, the early maladaptive abandonment schema and alexithymia were significantly correlated (*r* = 0.67, *p* < 0.001).

**Table 3:** Correlation analysis of gambling Motives, the early Maladaptive Schema, Alexithymia and Depression in men with gambling disorder

		1	2	3	4	5	6
1	GMQ_social	1					
2	GMQ_coping	0.19	1				
3	GMQ_enhance	0.12	0.49**	1			
4	Abandonment schema	0.01	0.07	− 0.26	1		
5	TAS-20	0.21	− 0.07	− 0.17	0.67**	1	
6	Depression	0.03	− 0.01	− 0.35*	0.72**	0.69**	1

GMQ = Gambling Motives Questionnaire; TAS-20 = Toronto Alexithymia Scale-20.** = *p* < .0.001; *p < 0.05

In the case of women (Table 4), significant and positive correlations were found for COP (*r* = 0.45, *p* < 0.001) and the early maladaptive abandonment schema (*r* = 0.43, *p* < 0.001) with depression, but no significant associations were observed between alexithymia and depression (*r* = 0.34, *p* = 0.129).

**Table 4:** Correlation analysis of Gambling Motives, the early Maladaptive Scheme, Alexithymia and Depression in women with gambling disorder

		1	2	3	4	5	6
1	GMQ_social	1					
2	GMQ_coping	0.30*	1				
3	GMQ_enhance	0.39**	0.25	1			
4	Abandonment scheme	0.15	0.25	0.15	1		
5	TAS-20	0.35	0.03	0.34	0.40	1	
6	Depression	0.14	0.45**	0.25	0.43**	0.34	1

GMQ = Gambling Motives Questionnaire; TAS-20 = Toronto Alexithymia Scale-20.** = *p* < 0.001; *p <  0.05

### Multiple Regression Analysis

Thirdly, multiple regression analysis was conducted to explore the predictive effect of gambling motives, abandonment schema and alexithymia on depression as an outcome variable (Tables 5 and 6). These analyses were conducted through the “Enter” method. That means that all the predictor variables (i.e., alexithymia, gambling motives and early abandonment schema) where inserted at the same time. Different analyses were conducted as a function of sex to explore which predictive variables explained depression in men and women. In men, the regression model was statistically significant (*R*^2^ = 0.706, *AR*^2^ = 0.620, *F* = 8.174, *p* < 0.001). As can be observed, alexithymia was the only predictive variable that showed statistically significant effects in male gamblers, indicating that the relationships with depression go beyond mere correlations (β = 0.95, *p* = 0.005).

**Table 5:** Multiple regression analysis of Gambling Motives, the early abandonment schema and Alexithymia as predictors of Depression in men with gambling disorder

Predictor variable	*B*	*β*	*t*	*p*	Variable criterion
GMQ_social	− 2.86	− 0.37	− 1.998	0.062	
GMQ_coping	0.26	0.07	0.376	0.712	
GMQ_enhance	0.37	0.10	0.489	0.631	Depression
Abandonment schema	0.01	0.01	0.018	0.986	
TAS-20	0.86	0.95	3.186	0.005	
					*R* = 0.840, *R*^2^ = 0.706, *A*R^2^ = 0.620, *F* = 8.174, *p* < 0.001

*B* = beta coefficient; *β* = beta standardised coefficient; *t* = t-Student; *p* = level of significance; *R*^2^ = coefficient of determination; *AR*^2^ = adjusted R-square; *F* = F of Snedecor; GMQ = Gambling Motives Questionnaire; TAS-20 = Toronto Alexithymia Scale-20

**Table 6:** Multiple regression of Gambling Motives, the early abandonment schema and Alexithymia as predictors of Depression in women with gambling disorder

Predictor variable	*B*	*β*	*t*	*p*	Variable criterion
GMQ_social	− 1.74	− 0.24	− 1.279	0.223	
GMQ_coping	1.38	0.41	2.297	0.039	
GMQ_enhance	0.01	0.00	0.002	0.998	Depression
Abandonment schema	1.15	0.49	2.530	0.025	
TAS-20	0.54	0.35	2.006	0.066	
					*R* = 0.821, *R*^2^ = 0.673, *A*R^2^ = 0.548, *F* = 5,361, *p* = 0.007

*B*= beta coefficient; *β* = beta standardised coefficient; *t* = t-Student; *p* = level of significance; *R*^2^ = coefficient of determination; *AR*^2^ = adjusted R-square; *F* = F of Snedecor; GMQ = Gambling Motives Questionnaire; TAS-20 = Toronto Alexithymia Scale-20

When the regression analyses model was conducted in women with GD, it also showed statistically significant results (*R*^2^ = 0.673, *AR*^2^ = 0.548, *F* = 5.361, *p* = 0.007). However, conversely to the results in men, alexithymia did not show a significant predictive effect on depression (β = 0.35, *p* = 0.066). However, the early maladaptive abandonment schema was the only variable that positively and significantly predicted depression in women (β = 0.49, *p* = 0.025), showing that a higher presence of abandonment schema indicates higher levels of depression.

### Mediation Analyses

**Fig. 1:**
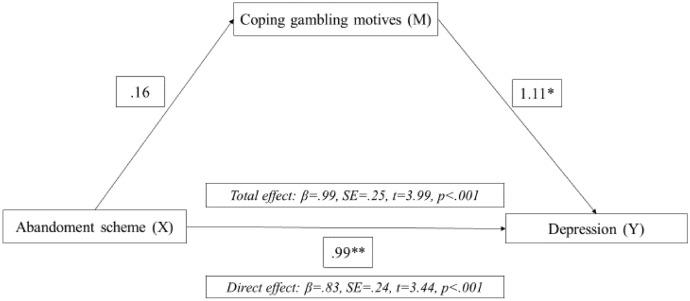
Mediation model of coping movies between the abandonment schema in depression in women with gambling disorder. *Note* **p > 0.001, *p > 0.05

**Fig. 2:**
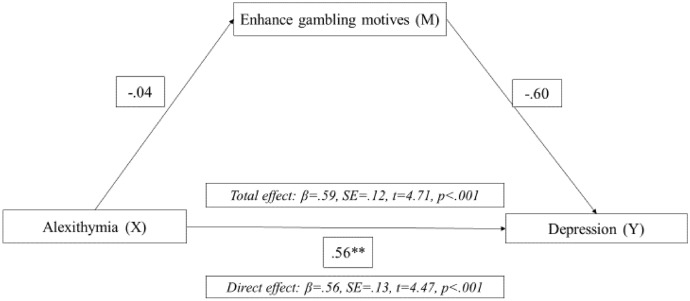
Mediation model of enhance motives between alexithymia in depression in men with gambling disorder. *Note* **p > 0.001, *p > 0.05

**Fig. 3:**
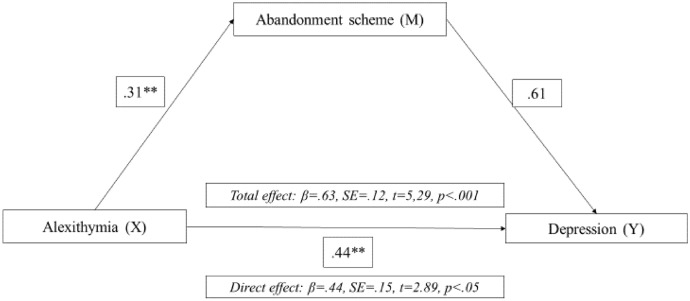
Mediation model of the abandonment schema between alexithymia oi depression in men with gambling disorder. *Note* **p > 0.001, *p > 0.05

Based on the results obtained through the correlation analysis (see Tables 3 and 4) and the regression analyses (see Tables 5 and 6), a mediational analysis was conducted to determine, on the one hand, whether in women, COP had a mediating effect on the relationship between abandonment schema and depression. On the other hand, in the sample of male gamblers, mediation analyses were conducted to determine whether ENH and/or abandonment schema had a mediating effect on the relationship between alexithymia and depression. In the case of women, the results confirmed that COP mediate the relationship between the predictor variable (abandonment schema) and depression (see Fig. 1). In the case of men, this mediating effect was also found in both mediation analyses, confirming that both ENH and abandonment schema mediate the relationship between alexithymia and depression (see Figs. 2 and 3).

## Discussion

The present study aimed to explore the predictive role of gambling motives, alexithymia and the early maladaptive abandonment schema on depressive symptomatology in men and women with Gambling Disorder (GD). To the best of our knowledge, no studies have explored the implication of the aforementioned variables in relation to GD, especially regarding women with GD. Previous research carried out by Macía et al. (2023) observed that the predictive variables of depressive symptoms in gamblers are not the same for both sexes. Their study suggests that alexithymia is likely to predict depression in male but not in female gamblers. Based on these findings, the present study goes one step further and explores, on the one hand, which variables may predict depressive symptomatology in women with GD and, on the other hand, which factors may mediate depressive symptoms in people with GD as a function of sex.

In this line, the first aim of the study was to explore differences between female and male gamblers in gambling motives (COP, ENH and SOC), maladaptive abandonment schema, alexithymia and depression. Despite the fact that based on previous literature, women could be expected to score higher on COP (Lelonek-Kuleta, 2021) and depression (Grant et al., 2012), differences were only found in alexithymia, where women's scores were higher.

Secondly, we examined possible sex differences in the relationship between gambling motives (COP, ENH and SOC), alexithymia, depression and early abandonment schema. Depression in men with GD was found to be significantly related to abandonment schema and alexithymia, and significantly but negatively related to ENH. In the case of female gamblers, depression significantly correlated with COP and abandonment schema. The relationship between abandonment schema and depressive symptomatology found in both sexes is consistent with a meta-analysis conducted by Tariq et al. (2021), which indicates that the Disconnection-Rejection dimension (which includes abandonment schema) is the best predictor of depression. This dimension is, in turn, one of the dimensions related to GD (Efrati et al., 2022). It is also interesting that depression is related to COP in female gamblers, whereas in males, ENH appears to have more weight, albeit negative, in depressive symptoms. These findings support previous evidence indicating that COP play a key role in the case of women with GD (Holdsworth et al., 2012; Macía et al., 2022). Moreover, COP are linked to chance games and to the need to escape from negative emotional states, which is common among female gamblers (Bonnaire et al., 2009; Lelonek-Kuleta, 2021). ENH have been related to sensation-seeking, strategic gambling typologies, impulsive traits, increased frequency of gambling and the expectation of fulfilling the need for arousal, all of which are more prevalent in male gamblers (Barrault et al., 2019; Stewart & Zack, 2008). Briefly, the findings of this study suggest that when faced with depressive symptoms, women are more likely to gamble with the expectation of alleviating negative affect, whereas men are more likely to deal with depression by increasing positive affect.

The third objective was to analyse the predictive role of gambling motives (COP and ENH), the early maladaptive abandonment schema and alexithymia on depression as a function of sex. The results indicate that depression in male gamblers is predicted by alexithymia and in female gamblers by early abandonment schema. Despite the fact that women with GD had higher alexithymia scores in the present study, alexithymia was not found to predict depressive symptomatology in women. Future studies could determine which variables interact with alexithymia in women with GD. However, the influence of the abandonment schema on depressive symptomatology in women with GD is noteworthy and may be revealing the influence of a relational aspect in women. In fact, in previous studies, abandonment has been related to fear of loneliness, which has been identified as a high-risk factor in female gamblers (Estévez et al., 2021; Mohammadkhani et al., 2017; Ozawa-de Silva & Parsons, 2020).

Lastly, based on the results obtained for women and men in the multiple regression analyses, mediation analyses were carried out. Regarding women with GD, we explored whether COP mediated the relationship between the early maladaptive abandonment schema and depressive symptomatology. In the case of the male gamblers, we examined whether ENH and/or early abandonment schema mediated the relationship between alexithymia and depressive symptoms. All mediation analyses were significant. That is, COP mediated the relationship between early abandonment schema and depressive symptoms in women, and ENH and abandonment schema mediated the relationship between alexithymia and depressive symptoms in men.

Regarding female gamblers, these findings are consistent with previous literature. It has been shown that the early abandonment schema predicts depression (Tariq et al., 2021) and that women tend to gamble to relieve their symptoms as an emotion- regulation or coping mechanism (Lelonek-Kuleta, 2021), which is, in fact, linked to GD severity (Stewart y Zack, 2008); and GD severity with depression (Richard et al., 2020; Wardell et al., 2015; Vaughan y Flack, 2022). Therefore, it is reasonable to have found evidence suggesting that COP mediates the relationship between abandonment schema and depression in women. In the case of male gamblers, the results of this study add to previous evidence supporting the predictive role of alexithymia on depressive symptomatology in men (Bonnaire et al., 2013; Macía et al., 2023). Despite this, this study provides additional evidence on the relationship between alexithymia and depression in men with GD, stressing the mediating effect of ENH and early abandonment schema. To our knowledge, these findings provide further insight into the clinical implications of gender issues in GD. However, further studies are still needed to deepen the clinical profile of women with GD.

### Limitations

This study has some limitations. In the first place, as this is a cross-sectional study, causal relationships cannot be established. Therefore, it would be advisable to carry out longitudinal studies in the future. Secondly, the sample size is small, so the generalisation of the results should be made with caution. However, it is difficult to find samples of women with GD, and very few studies include them, so this study will likely contribute to further knowledge. In addition, the sample was recruited through associations for the rehabilitation of GD. Therefore, its clinical profile might differ from that of other clinical samples (e.g., untreated individuals, public hospitals, younger samples, private therapeutic centres, etc.). Finally, the results of the present study are based on sex differences (i.e., women or men), so more gender-specific studies are needed in the future (E.g. the choice to express their gender identity, including possibilities such as trans/non-binary/other…).

## Conclusion

In conclusion, this study adds evidence of the differences between men and women with GD. Depression is predicted by alexithymia in men with GD and by the early maladaptive abandonment schema in women with GD. Furthermore, COP has been found to mediate the relationship between early abandonment schema and depression in women. In the case of male gamblers, the results suggest that abandonment schema and ENH mediate the relationship between alexithymia and depression.

Bearing this information in mind may be relevant for establishing sex-specific therapeutic strategies. In addition, it would be important to consider the results for preventive issues. In this regard, structuring of psychoeducational programs aimed at adolescents and parents could be beneficial, especially in the field of primary prevention. Parallelly, implementing psychoeducational programmes for couples, children or parents who have family members with GD could also be of interest in order to make the treatment as integrative as possible.

To sum up, the existing body of research on GD was based on predominantly male samples. This implies that such findings may not be generalisable to samples of women with GD. Therefore, we stress the importance of continuing research on female gamblers to provide high-quality psychological assistance for women tailored to their needs.

## Data Availability

The datasets generated during and/or analysed during the current study are not publicly available due to confidentially.
